# The histone deacetylase HDAC1 positively regulates Notch signaling during *Drosophila* wing development

**DOI:** 10.1242/bio.029637

**Published:** 2018-02-01

**Authors:** Zehua Wang, Jialan Lyu, Fang Wang, Chen Miao, Zi Nan, Jiayu Zhang, Yongmei Xi, Qi Zhou, Xiaohang Yang, Wanzhong Ge

**Affiliations:** 1 Division of Human Reproduction and Developmental Genetics, The Women's Hospital, Zhejiang University School of Medicine, Hangzhou, Zhejiang 310058, China; 2 Institute of Genetics and Department of Genetics, Zhejiang University School of Medicine, Hangzhou, Zhejiang 310058, China; 3 College of Life Sciences, Zhejiang University, Hangzhou, Zhejiang 310058, China; 4 Life Sciences Institute, Zhejiang University, Hangzhou, Zhejiang 310058, China

**Keywords:** Notch, HDAC1, Atrophin, *Drosophila* wing development

## Abstract

The Notch signaling pathway is highly conserved across different animal species and plays crucial roles in development and physiology. Regulation of Notch signaling occurs at multiple levels in different tissues and cell types. Here, we show that the histone deacetylase HDAC1 acts as a positive regulator of Notch signaling during *Drosophila* wing development. Depletion of *HDAC1* causes wing notches on the margin of adult wing. Consistently, the expression of Notch target genes is reduced in the absence of HDAC1 during wing margin formation. We further provide evidence that HDAC1 acts upstream of Notch activation. Mechanistically, we show that HDAC1 regulates Notch protein levels by promoting Notch transcription. Consistent with this, the HDAC1-associated transcriptional co-repressor Atrophin (Atro) is also required for transcriptional activation of Notch in the wing disc. In summary, our results demonstrate that HDAC1 positively regulates Notch signaling and reveal a previously unidentified function of HDAC1 in Notch signaling.

## Abstract (precis)

**Summary:** The histone deacetylase HDAC1 acts as a positive regulator of Notch signaling during *Drosophila* wing development, and its depletion causes wing notches on the margin of adult wing.

## INTRODUCTION

The Notch pathway is an evolutionarily conserved signaling cascade present in most multicellular organisms, and plays important roles during development and adult life (Bray, 2006; Andersson et al., 2011; Guruharsha et al., 2012). Notch signaling regulates various biological processes, including cell fate determination, cell proliferation, differentiation and apoptosis (Bray, 2006; Andersson et al., 2011; Guruharsha et al., 2012). Misregulation of Notch activity has been associated with many diseases, such as cancers and neurological disorders (Alberi et al., 2013; Aster et al., 2017). In the canonical Notch signaling, Notch is a transmembrane receptor expressed on the cell surface (Bray, 2006; Andersson et al., 2011; Guruharsha et al., 2012). Upon binding to its ligands (Delta/Serrated/LAG-2, DSL), Notch gets activated and undergoes a sequence of proteolytic cleavage, resulting in the generation of the Notch intracellular domain (NICD) (Bray, 2006; Andersson et al., 2011; Guruharsha et al., 2012). The released NICD is translocated from the cell membrane to the nucleus where it interacts with the DNA-binding transcription factor CSL (CBF1/Suppressor of Hairless/LAG-1) (Bray, 2006; Andersson et al., 2011; Guruharsha et al., 2012). The association of CSL with NICD promotes transcription of target genes by forming a transcriptional activator complex, which contains Mastermind (MAML in mammals, Mastermind in flies) and histone acetyltransferase p300/CBP (Bray, 2006; Andersson et al., 2011; Borggrefe and Liefke, 2012; Guruharsha et al., 2012). Notch signaling is tightly regulated at multiple levels, including Notch protein modification, trafficking, recycling and degradation (Bray, 2006; Andersson et al., 2011; Guruharsha et al., 2012). However, it remains largely unexplored how Notch signaling is regulated at the level of Notch receptor transcription.

Histone deacetylases (HDACs) are one class of enzymes which normally control the acetylation status of histones through catalyzing the removal of the acetyl groups (Struhl, 1998). Deacetylation of histones by HDACs promotes chromatin condensation and thereby represses transcription (Haberland et al., 2009). Although the roles of HDAC in gene silencing are well established, HDAC-mediated gene activation has also been reported in various contexts (Nusinzon and Horvath, 2003; Chang et al., 2004; De Nadal et al., 2004; Sharma et al., 2007; Dovey et al., 2010). In addition to the histone substrate, HDACs also act on non-histone protein substrates, including transcription factors and chaperons, and regulate their activities (Choudhary et al., 2009; Johnson and Kornbluth, 2012). Human HDACs are divided into four classes based on their homology to yeast proteins (Yang and Seto, 2008; Haberland et al., 2009). Among them, the best studied HDACs are the class I enzymes HDAC1 and HDAC2, which share homology with the yeast protein Rpd3 (Yang and Seto, 2008; Haberland et al., 2009). HDAC1/2 and Rpd3 are generally associated with large transcriptional repressor complexes, such as Sin3, NuRD, and CoREST complexes, and have regulatory functions in various signaling pathways (Kelly and Cowley, 2013).

In the absence of Notch activation, it is thought that CSL functions as a suppressor by associating with transcriptional repressor proteins, such as Hairless in flies, SMRT and histone deacetylase HDAC1 in mammals (Morel et al., 2001; Barolo et al., 2002; Borggrefe and Liefke, 2012; Schwanbeck, 2015; Borggrefe and Oswald, 2016). With histone modification activity, the repressor complex maintains a repressive chromatin environment at different Notch target gene loci (Borggrefe and Liefke, 2012; Schwanbeck, 2015; Borggrefe and Oswald, 2016). It has been proposed that HDAC1 functions as a negative regulator of Notch signaling (Borggrefe and Liefke, 2012; Schwanbeck, 2015; Borggrefe and Oswald, 2016). Consistent with this view, HDAC1 interacts with CBF1 in mammalian cells, and treatment of TSA, a HDAC1 inhibitor, derepresses Notch target gene ESR-1 expression (Kao et al., 1998). In zebrafish, it has also been shown that two Notch target genes, *her6* and *her4*, are upregulated in *HDAC1* mutants (Cunliffe, 2004; Yamaguchi et al., 2005). In mice, overexpression of HDAC1 or HDAC2 enhances the repression of Notch target gene *Hey2* expression mediated by overexpression of *Zeb2* (Wu et al., 2016). However, genetic studies for the interaction between HDAC1 and Notch signaling are still limited to certain biological processes. It is unknown whether HDAC1 has additional roles in Notch signaling, except for the formation of CSL-mediated transcriptional repressor complex.

Here, we use *Drosophila* wing imaginal disc as a model system to address the function of HDAC1 in regulating the Notch signaling pathway. Unexpectedly, we find that loss of *HDAC1* function causes wing notching and reduces Notch target gene expression. Furthermore, we provide evidence that HDAC1 positively affects Notch signaling by promoting *Notch* transcription. Consistently, transcriptional activation of Notch also requires the activity of Atrophin (Atro), a transcriptional co-repressor which has been reported to directly interact with HDAC1 (Wang et al., 2008; Zhang et al., 2013). Together, our data indicate that the histone deacetylase HDAC1 acts as a positive regulator of Notch signaling during *Drosophila* wing development.

## RESULTS

### HDAC1 regulates Notch signaling in the *Drosophila* wing

**Fig. 1.**
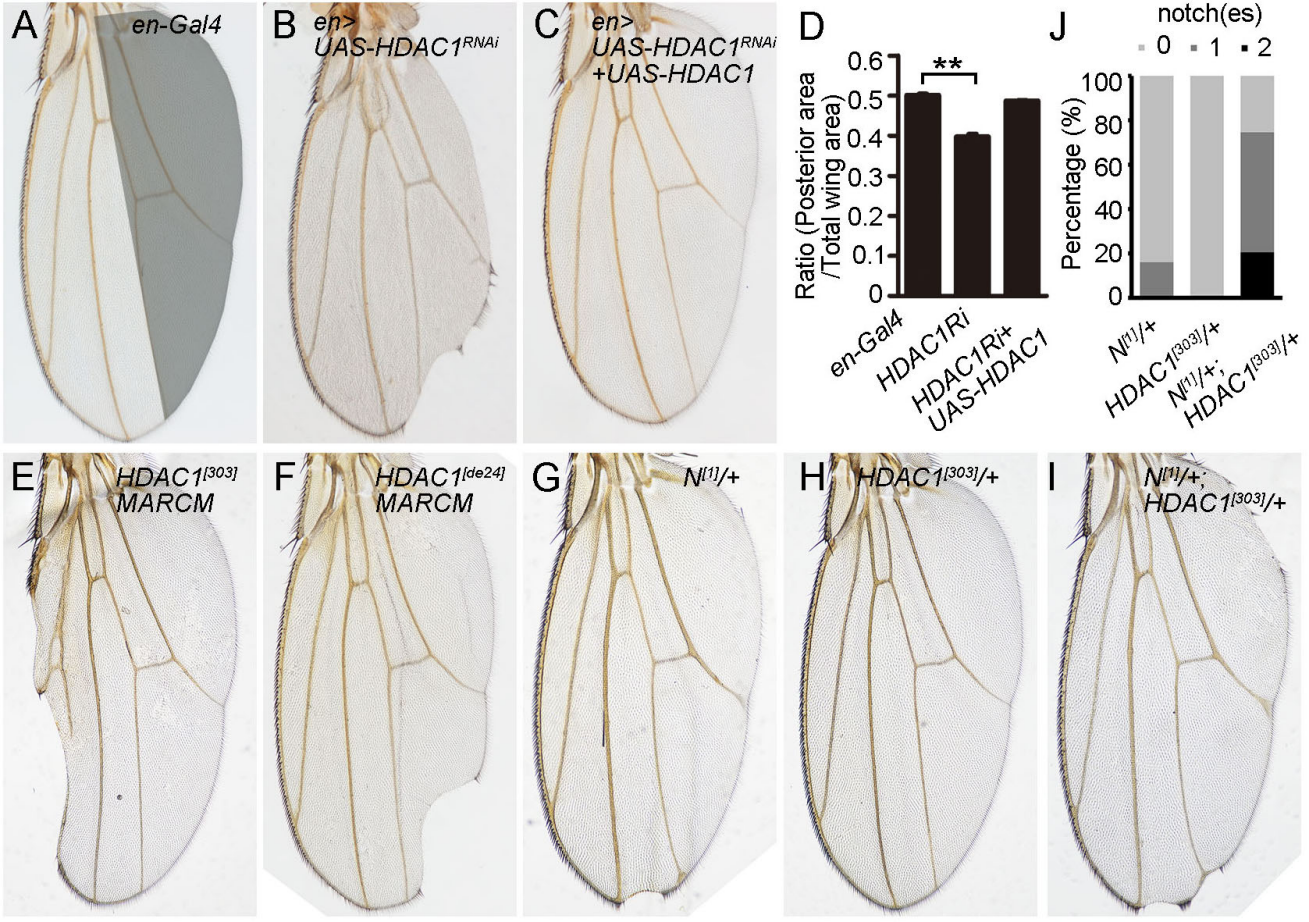
**Loss of HDAC1 causes wing notching in *Drosophila.*** (A-C) Adult wings from flies expressing the following transgenes under the control of *en-Gal4*: (A) control, (B) *UAS-HDAC1 RNAi*, (C) *UAS-HDAC1 RNAi* and *UAS-HDAC1*. Depletion of *HDAC1* causes loss of wing margin tissue. The posterior half of the wing is marked by grey shadow. (D) Quantification of wing size of flies with the indicated genotypes. The ratio was defined by the posterior wing area divided by total wing area. *n*=5 for each genotype. **P<0.01. (E,F) Adult wings from flies bearing *HDAC1^[303]^* (E) or *HDAC1^[def24]^* (F) MARCM mutant clones exhibit wing notches. (G-I) Adult wings from flies with the indicated genotypes. Wings from *N^[1]^/+; HDAC1^[303]^/+* doubly heterozygotes display notched wing margin with higher frequency compared to *N^[1]^*/+ heterozygotes. (J) Quantification of Notch wing phenotype in *N^[1]^/+* (*n*=62), *HDAC1^[303]^/+*(*n*=75) and *N^[1]^/+; HDAC1^[303]^/+* (*n*=122) flies.

Loss of function of *HDAC1* is lethal at early larval stages, suggesting that HDAC1 plays an essential role during *Drosophila* development (Zhu et al., 2008). To examine the function of HDAC1 in various tissues and at different developmental stages, we began our study by analyzing the effect of knocking down *HDAC1* activity using RNAi with various Gal4 drivers on wing development. Knockdown of *HDAC1* using the *UAS-HDAC1-RNAi* line in the developing wing under the control of *en-Gal4* caused wing patterning and growth defects, including wing notches, disorganized vein pattern, ectopic sensory bristles in the distal part between L4 and L5, and a reduction of the posterior size of the wing (Fig. 1A,B,D). As the targeting region of *UAS-HDAC1-RNAi* is within the 3′UTR of *HDAC1* mRNA, we used the *UAS-HDAC1* transgene to perform the rescue experiment (Tea et al., 2010; Ni et al., 2011). Overexpression of *HDAC1* was able to rescue the RNAi phenotype, confirming that these wing defects were due to specific knockdown of *HDAC1*, but not the result of off-target effects (Fig. 1C,D). Furthermore, antibody staining revealed that *HDAC1* RNAi led to a significant reduction of HDAC1 protein levels in wing discs, indicating the high efficiency of the RNAi line we used (Fig. S1A,A′). Similar to the *en-Gal4* driver, RNAi of *HDAC1* by *ptc-Gal4*, *vg-Gal4* or *nub-Gal4* all led to the notched wing phenotype as well as other patterning defects (Fig. S2A-F).

To verify these phenotypes, we generated MARCM clones for two *HDAC1* mutant alleles, *HDAC1^[303]^* and *HDAC1^[def24]^*, in the larval stage and later analyzed the adult wing morphology. *HDAC1^[303]^* is a hypomorphic allele associated with a point mutation in a highly conserved region of the protein, which is thought to be required for its deacetylase activity (Mottus et al., 2000). *HDAC1^[def24]^* is a loss-of-function mutant allele in which part of the amino terminal coding region is deleted (Mottus et al., 2000). Reduction or loss of *HDAC1* activity indeed caused notches on the adult wing margin (Fig. 1E,F). The notched wing phenotype in the *HDAC1* RNAi and mutant flies is typical of a reduction of Notch signaling, which suggests that *Drosophila* HDAC1 may positively regulate Notch signaling. This was further confirmed by the genetic interaction analysis between *HDAC1* and *Notch* mutants. For this analysis, we examined the effect of removal of one copy of *HDAC1* in the *Notch* mutant background. Flies heterozygous for the *Notch* null mutant allele *N^[1]^* showed thickened wing vein and the classic notched wing phenotype in ∼16% of the wings (Fig. 1G,J). Each of them had only one notch on the wing margin (Fig. 1G,J). Reducing the dose of *HDAC1* by one copy enhanced the notched wing phenotype, as 80% of the wings from *N^[1]^/+; HDAC1^[303]^/+* flies displayed one or two notches on the wing margin and had an increase in the severity of the phenotype (Fig. 1H-J).

Taken together, these data demonstrate that loss of *HDAC1* leads to reduced Notch signaling and HDAC1 might act as a positive regulator of the Notch pathway during *Drosophila* wing development.

### HDAC1 promotes Notch target gene expression during wing margin formation

**Fig. 2.**
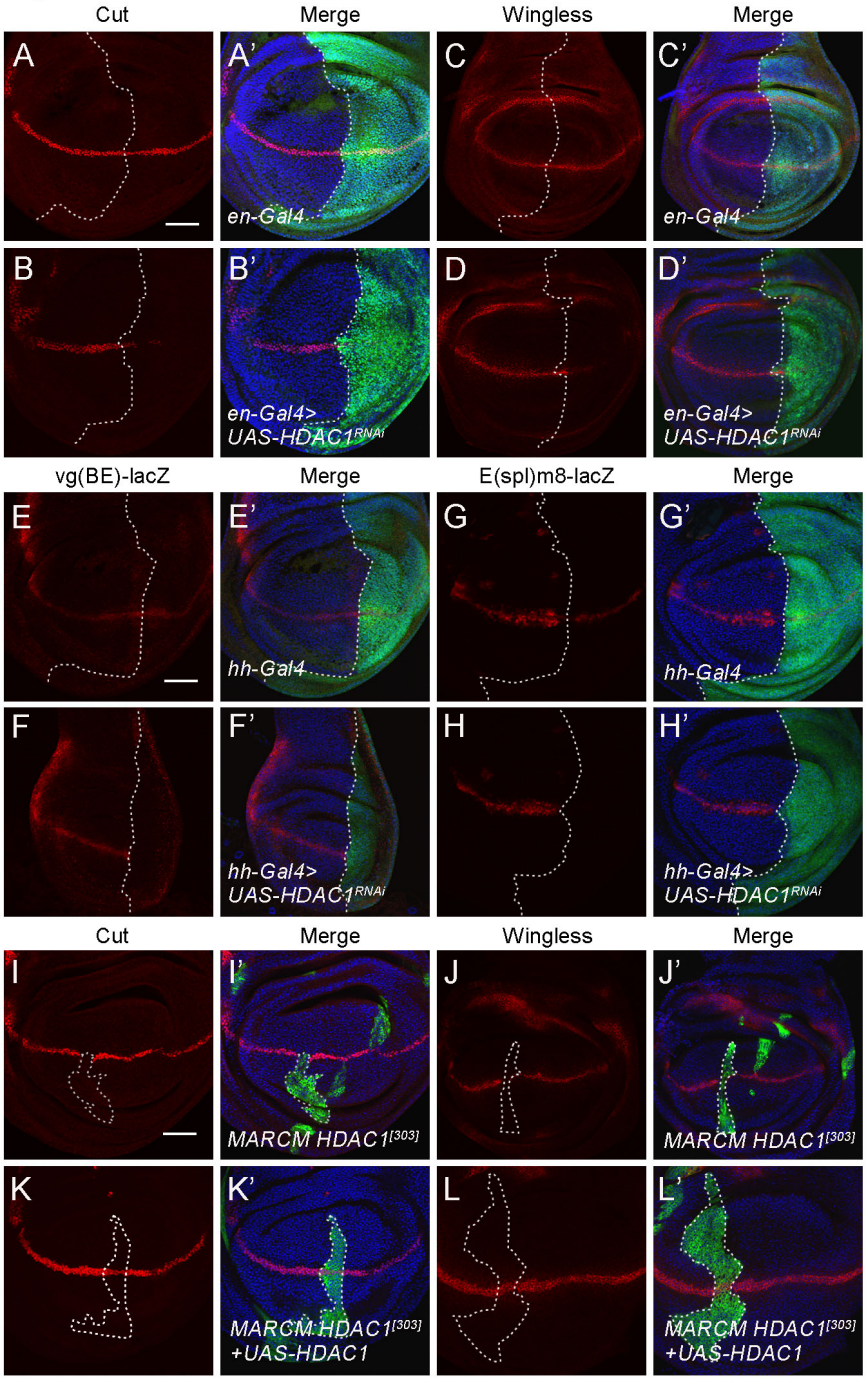
**HDAC1 is required for Notch target gene expression.** (A-D′) RNAi knockdown of *HDAC1* with *en-Gal4* reduces Cut (A-B′) and Wingless (C-D′) protein levels in the posterior half of the wing disc. GFP marks the expression domain of *en-Gal4*. (E-E′) Expression pattern of the *vg(BE)-lacZ* reporter gene in a control wing imaginal disc. (F-F′) Knockdown of *HDAC1* by RNAi with *hh-Gal4* eliminates *vg(BE)-lacZ* expression in the posterior compartment of the wing disc. (G-G′) Expression pattern of the *E(spl)m8-lacZ* reporter gene in a control wing imaginal disc. (H-H′) Knockdown of *HDAC1* by RNAi with *hh-Gal4* reduces the level of *E(spl)m8-lacZ* expression in the posterior compartment of the wing disc. Discs in E-H′ were stained with anti-β-Gal and anti-GFP. DNA was stained with DAPI. Posterior cells are marked by GFP. (I-J′) MARCM clones of *HDAC1^[303]^* in the wing disc results in a decrease of Cut (I,I′) and Wingless (J,J′) protein levels. (K-L′) Overexpression of *HDAC1* in MARCM clones of *HDAC1^[303]^* in the wing disc restores Cut (K,K′) and Wingless (L,L′) protein levels. Mutant clones are marked by the presence of GFP. Scale bars: 50 μm.

To further investigate the relationship between HDAC1 and the Notch pathway, we expressed the *HDAC1* RNAi transgene in wing imaginal discs and assayed the expression of multiple Notch target genes, including *Cut*, *Wingless*, *vg* and *E(spl)m8*. In the wild-type wing disc, Cut and Wingless are induced in a narrow strip of cells at the dorsal ventral compartment boundary in response to Notch signaling (Fig. 2A,C). *en-Gal4*-driven expression of *HDAC1* RNAi reduced Cut and Wingless protein levels in the posterior compartment of the wing disc (Fig. 2A-D′). The *vg* boundary enhancer, *vg(BE)-lacZ*, is a more sensitive and specific reporter for Notch signaling. In wild-type flies carrying the *vg(BE)-lacZ* reporter, lacZ expression was mainly observed along the dorsal ventral boundary of the wing disc (Fig. 2E). In contrast, depletion of *HDAC1* by RNAi with *hh-Gal4* completely eliminated *vg(BE)-lacZ* expression in the posterior compartment (Fig. 2E-F′). In addition, we examined expression of the Notch target E(spl)m8, which is also induced by Notch and expressed along the dorsal ventral boundary (Fig. 2G). Expression of *E(spl)m8-lacZ*, an *E(spl)m8* reporter, was abolished by *HDAC1* RNAi (Fig. 2G-H′). As transcription of *vg* and *E(spl)m8* was dependent on the binding between their regulatory sequences and Suppressor of Hairless [Su(H)], these data suggest that HDAC1 is required for Su(H)-mediated Notch activation. Similarly, Cut and Wingless protein levels were reduced in *HDAC1^[303]^* mutant MARCM clone cells along the DV boundary (Fig. 2I-J′). Overexpression of *HDAC1* was able to restore Cut and Wingless expression levels in *HDAC1^[303]^* mutant clone cells (Fig. 2K-L′). These results further confirm that HDAC1 is required for Notch target gene expression.

Collectively, these findings support the notion that HDAC1 promotes Notch downstream target gene expression during wing margin formation, which is consistent with the analysis of adult phenotypes.

### HDAC1 acts upstream of Notch activation

**Fig. 3.**
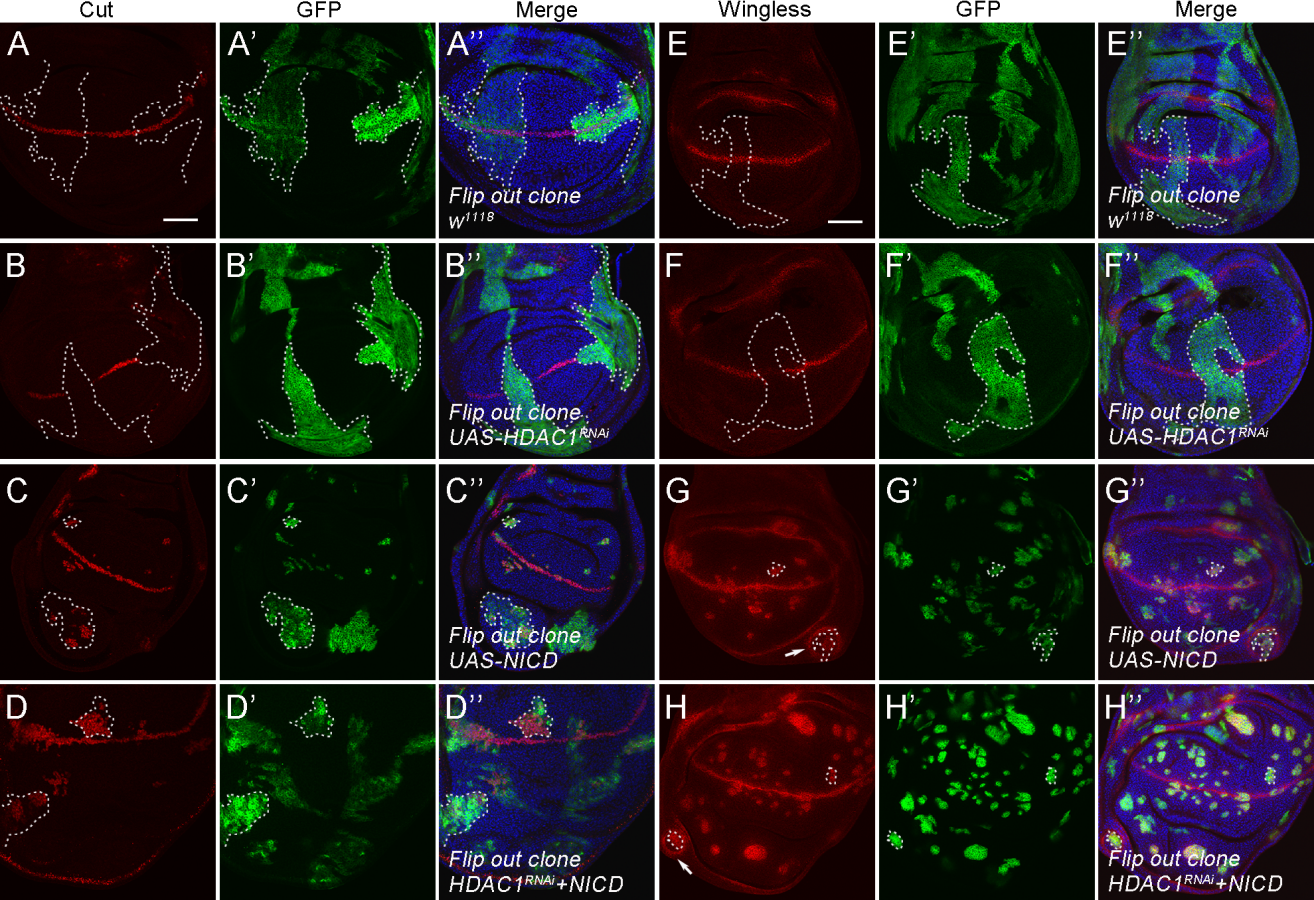
**HDAC1 acts upstream of Notch activation.** (A-D″) Co-expression of *HDAC1* RNAi with the active form of Notch (NICD) does not suppress NICD-induced ectopic Cut expression. Wing discs from animals expressing the following transgenes with the Flip-out system: (A) control, (B) *UAS-HDAC1 RNAi*, (C) *UAS-NICD*, (D) *UAS-NICD* and *UAS-HDAC1 RNAi*, stained with anti-Cut and anti-GFP. DNA was stained with DAPI. Clones are marked by dashed lines. (E-H″) Co-expression of HDAC1 RNAi with the active form of Notch (NICD) does not suppress NICD-induced ectopic Wingless expression. Wing discs from animals expressing the following transgenes with the Flip-out system: (A) control, (B) *UAS-HDAC1 RNAi*, (C) *UAS-NICD*, (D) *UAS-NICD* and *UAS-HDAC1 RNAi*, stained with anti-Wingless and anti-GFP. DNA was stained with DAPI. Clones are marked by dashed lines. Arrows indicate cells with non-autonomous Wingless expression. Scale bars: 50 μm.

To determine at which step HDAC1 might function in the Notch pathway, we performed the genetic epistasis analysis. Activation of Notch by its ligands leads to the release of the Notch intracellular domain NICD (Bray, 2006; Andersson et al., 2011). The transcriptionally active NICD enters the nucleus and promotes target gene transcription (Bray, 2006; Andersson et al., 2011). Ectopic expression of NICD results in misexpression of target genes. We examined whether *HDAC1* RNAi was able to suppress the elevated Notch target gene expression caused by overexpression of NICD. To this purpose, we generated clones in the wing disc using the Flip-out approach and performed antibody staining. Consistent with our previous results, clonal expression of *HDAC1* RNAi reduced Cut expression along the DV boundary, while the wild-type control clones showed normal Cut expression (Fig. 3A-B″). In both dorsal and ventral compartments of the wing disc, Cut expression was induced in clones where NICD was ectopically expressed (Fig. 3C-C″). We found that this NICD-dependent Cut expression was not affected by co-expression of *HDAC1* RNAi (Fig. 3D-D″). In addition to Cut expression, we also examined the expression of Wingless. Reduced Wingless expression was observed in *HDAC1* RNAi clones as compared to its normal expression in wild-type control clones (Fig. 3E-F″). Ectopically expressed NICD caused the upregulation of Wingless expression in the clones (Fig. 3G-G″). However, NICD-induced Wingless expression was not suppressed by *HDAC1* RNAi (Fig. 3H-H″). As reported previously, NICD overexpression can induce non-autonomous cell proliferation and Wingless expression outside of NICD-expressing clones (Giraldez and Cohen, 2003) (arrow in Fig. 3G-G″). We found that expression of *HDAC1* RNAi did not affect Wingless expression either in cells adjacent to NICD-expressing clones (arrow in Fig. 3H). These results together suggest that HDAC1 functions upstream of Notch activation.

### HDAC1 affects Notch protein levels and regulates Notch transcription

**Fig. 4.**
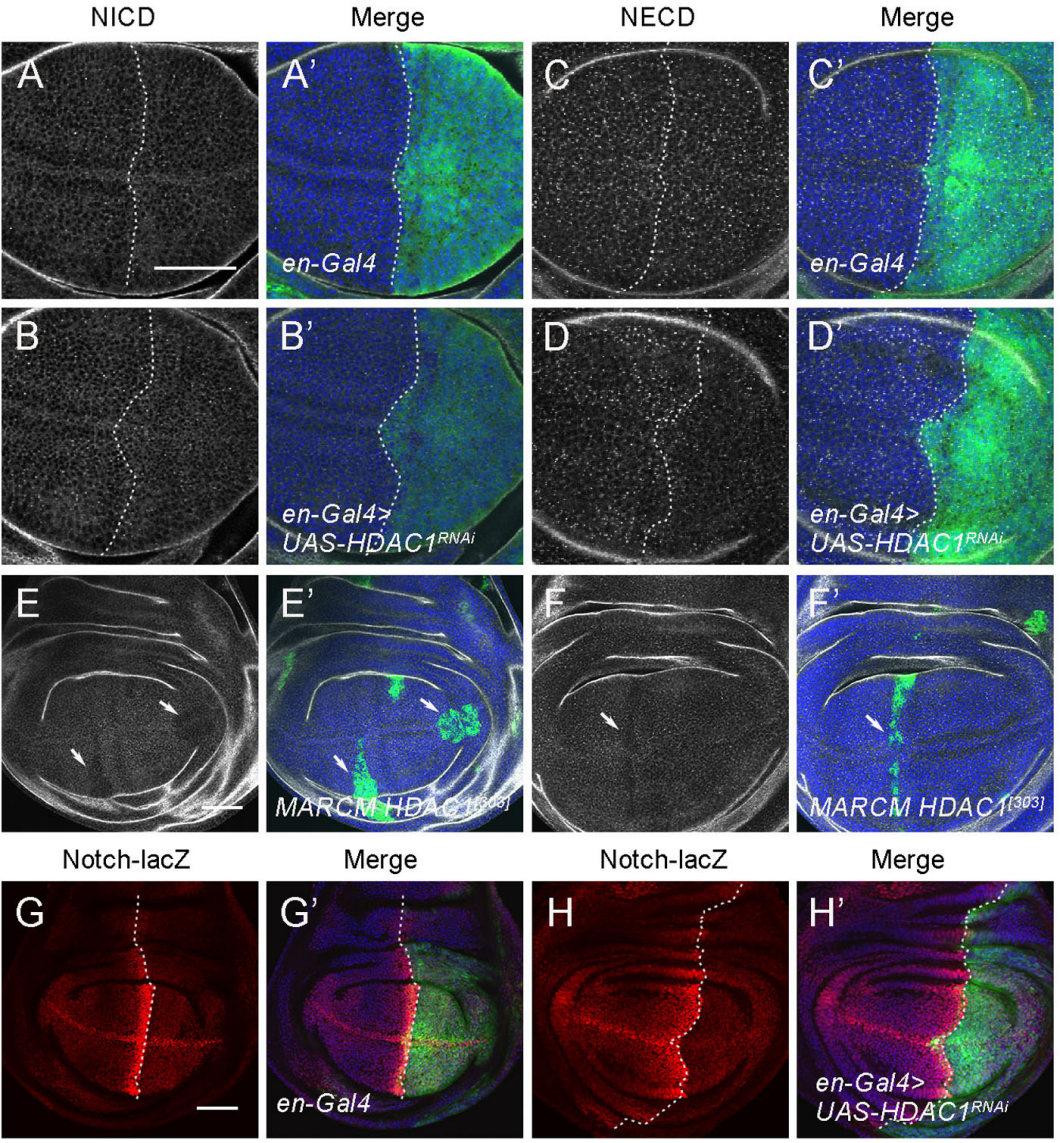
**HDAC1 regulates Notch protein levels and Notch transcription.** (A-D′) RNAi knockdown of *HDAC1* with *en-Gal4* reduces NICD (A-B′) and NECD (C-D′) protein levels in the posterior half of the wing disc. GFP marks the expression domain of *en-Gal4*. (E-F′) MARCM clones of *HDAC1^[303]^* in the wing disc results in a decrease of NICD (E-E′) and NECD (F-F′) protein levels. Mutant clones are marked by presence of GFP. Arrows indicate the clone area. (G-G′) Expression pattern of the *Notch-lacZ* reporter gene in a control wing imaginal disc. (H-H′) Knockdown of *HDAC1* by RNAi with *en-Gal4* reduces *Notch-lacZ* expression in the posterior compartment of the wing disc. Scale bars: 50 μm.

Regulation of Notch protein activity occurs at multiple levels, including transcriptional control, posttranscriptional modifications, vesicle transport and protein degradation (Andersson et al., 2011). To gain insight into the mechanism of Notch regulation by HDAC1, we examined the effects of loss of *HDAC1* on endogenous Notch protein distribution in the wing imaginal disc. Endogenous Notch protein is expressed ubiquitously throughout the wing disc. We performed the staining using antibodies that recognize both Notch intracellular domain (anti-NICD) as well as the extracellular domain (anti-NECD). Compared with the controls, RNAi of *HDAC1* by *en-Gal4* slightly reduced the levels of Notch protein in the posterior compartment (Fig. 4A-D′). We further confirmed these results in *HDAC1^[303]^* MARCM mutant clones. NICD and NECD protein levels were clearly reduced in *HDAC1^[303]^* mutant cells compared with the surrounding wild type cells (Fig. 4E-F′, arrows indicate the clone areas).

The reduction of Notch protein levels could be due to a decreased *Notch* expression or Notch degradation. To discriminate these two possibilities, we made use of a P-lacZ insertion at Notch locus and analyzed Notch expression (de Celis et al., 1997; Zacharioudaki and Bray, 2014). Knockdown of *HDAC1* with *en-Gal4* resulted in a clear reduction of *Notch-lacZ* expression in the posterior region (Fig. 4G-H′). Thus, the reduction of Notch protein was due to the reduced transcription of the *Notch* gene in *HDAC1* depletion cells.

We next examined whether overexpression of *HDAC1* was sufficient to drive Notch transcription. For this purpose, we ectopically expressed *HDAC1* using *en-Gal4* in the posterior compartment of the wing disc and analyzed *Notch-lacZ* expression. Overexpression of *HDAC1* had no effects on the expression of *Notch-lacZ* (Fig. S3A-B′). In addition, the expression of two Notch target genes, Cut and Wingless, was not altered when *HDAC1* was overexpressed (Fig. S3C-F′), indicating that HDAC1 overexpression might not affect Notch signaling in the wing disc. Based on these results, we concluded that HDAC1 functions to promote Notch signaling by regulating the transcription of the Notch gene.

### Regulation of Notch transcription by the HDAC1-associated transcriptional co-repressor Atro

**Fig. 5.**
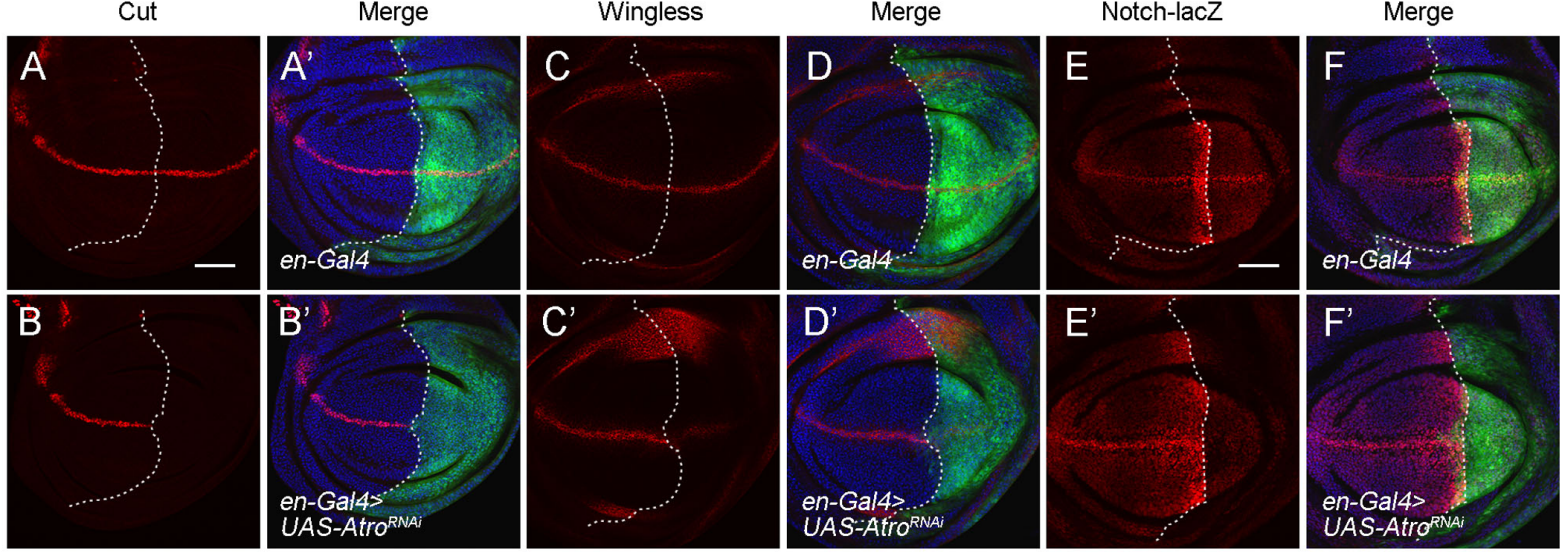
**Atro is required for Notch target gene expression and Notch transcriptional activation.** (A-D′) Knockdown of *Atro* by RNAi with *en-Gal4* reduces Cut (A-B’) and Wingless (C-D′) protein levels in the posterior half of the wing disc. (E-F′) Knockdown of *Atro* by RNAi with *en-Gal4* reduces *Notch-lacZ* expression in the posterior compartment of the wing disc. GFP marks the expression domain of *en-Gal4*. Scale bars: 50 μm.

HDAC1 can interact with the transcriptional co-repressor Atro to control Hh signaling in the *Drosophila* wing disc (Zhang et al., 2013). The association between HDAC1 and Atro has also been reported to function to determine cell fates in *Drosophila* (Wang et al., 2008). *Drosophila* Atro functions in multiple developmental processes and has recently been shown to regulate several developmental signaling pathways, including Notch signaling (Zhang et al., 2002; Yeung et al., 2017). Loss of *Atro* causes wing notches and other pattering defects, which are similar to the phenotype we observed for *HDAC1* mutants (Zhang et al., 2002; Yeung et al., 2017). The phenotypical similarity and physical association between Atro and HDAC1 prompted us to test whether Atro also regulates Notch transcription. To this end, we performed RNAi knockdown analysis in the wing disc. We first confirmed that RNAi of *Atro* by *en-Gal4* led to the downregulation of Notch target gene expression, including Cut and Wingless (Fig. 5A-D′). This is consistent with a previous study and also suggests that the RNAi line we used is efficient to reduce Atro activity (Yeung et al., 2017). Next, we examined the expression of *Notch-lacZ* in *Atro* knockdown wing discs. Decreased expression of *Notch-lacZ* was evident in the posterior compartment of the wing disc (Fig. 5E-F′). These results demonstrate that Atro, similar to HDAC1, is required for transcriptional activation of Notch receptor gene during *Drosophila* wing development.

## DISCUSSION

The data presented here describe the histone deacetylase HDAC1 as a positive regulator of the Notch signaling pathway in *Drosophila*. Although *Drosophila* HDAC1 acts broadly to regulate the activity of many signaling pathways, including Hippo, JNK and Hh, our data support a specific interaction between HDAC1 and Notch activity during wing development (Zhang et al., 2013, 2016).

HDAC1 has been previously reported to repress Notch signaling in different contexts (Cunliffe, 2004; Yamaguchi et al., 2005; Wu et al., 2016). It is generally accepted that CSL can recruit the HDAC1 transcriptional co-repressor complex to modify chromatin structure for target gene silencing before Notch activation (Schwanbeck, 2015; Borggrefe and Oswald, 2016). Contrary to this, we show that loss of *HDAC1* decreases Notch signaling, suggesting an additional role of HDAC1 in regulating the Notch pathway. Reduction of Notch transcription in *HDAC1* depletion wing tissues reveals a potential mechanism by which HDAC1 activates Notch transcription. It is highly possible that HDAC1 has a direct positive role at the Notch gene locus, which could be the direct regulation of histone deacetylation by HDAC1. Alternatively, HDAC1 affects Notch transcription through deacetylation of other transcription factors present at the promoter region of Notch gene. The involvement of HDAC1 in gene activation has been suggested in various studies, although the molecular mechanism behind this remains largely unknown (Dovey et al., 2010).

We show clearly that HDAC1 regulates Notch transcription. However, other mechanisms mediated by HDAC1 in contribution to Notch activation might co-exist. For instance, HDAC1 could repress expression of other negative regulators of Notch signaling. In addition, HDAC1 could act independently of the transcription, and directly deacetylate a key component of the Notch pathway. As HDAC1 has been shown to be associated with Su(H) in *Drosophila* cells, it may deacetylate Su(H) or other factors in the activator complex, leading to the stabilization of these proteins and Notch activation (Moshkin et al., 2009). Further studies are needed to test these possibilities.

Epigenetic regulation of the Notch pathway involves a transition of the suppressor to the activator complex at Notch target gene loci (Schwanbeck, 2015). Several chromatin modifying factors are involved in this process, including Kdm5A, PRC1, PRC2, Sirt1, LSD1 and HDAC1 (Borggrefe and Liefke, 2012; Schwanbeck, 2015; Borggrefe and Oswald, 2016). Previous studies focus on the repressing role of these proteins at Notch target gene loci, and conclude that these factors are negative regulators of the Notch pathway (Borggrefe and Liefke, 2012; Schwanbeck, 2015; Borggrefe and Oswald, 2016). For example, it has been reported that *Drosophila* Sirt1 and LSD1 function to regulate Notch target gene expression and suppress Notch signaling (Mulligan et al., 2011). However, a recent study demonstrates that Sirt1 has a positive role on Notch activation by controlling the deacetylation of Su(H) (Horvath et al., 2016). Furthermore, Two other HDAC1-associated proteins, CoRest and Brms1, have been reported to function as positive regulators of Notch signaling in *Drosophila* (Domanitskaya and Schupbach, 2012; Zhang et al., 2014). Interestingly, Brms1 is required for transcriptional activation of Notch. We show here that both HDAC1 and Atro can promote Notch transcription. Taken together, it is conceivable that a large protein complex containing HDAC1, Atro and Brms1 may play a crucial role in controlling Notch receptor transcription. These studies also demonstrate that the regulation of Notch signaling by these chromatin modifying factors are more complicated than previously thought. Such complex and tight regulation of Notch signaling are important during development.

## MATERIALS AND METHODS

### *Drosophila* stocks

The following fly stocks were used: *w^1118^*, *UAS-HDAC1^RNAi^* (THU0695, Tsinghua Fly Center), *UAS-Atro^RNAi^* (THU1153, Tsinghua Fly Center), *UAS-HDAC1.V5* (BL32241), *UAS-NICD*, *HDAC1^[def24]^ FRT2A FRT82B/TM6B*, *Tb* (BL32239), *HDAC1^[303]^ FRT2A FRT82B/TM6B*, *Tb* (BL32240), *N^1^* (BL6873), *en-Gal4 UAS-GFP*/Cyo, *vg-Gal4*, *nub-Gal4*, *ptc-Gal4*, *vg(BE)-lacZ/Cyo; hh-Gal4 UAS-GFP/TM6B*, *E(spl)m8-lacZ/Cyo; hh-Gal4 UAS-GFP/TM6B*, *Notch-lacZ; en-Gal4 UAS-GFP*/Cyo, *MARCM2A (hsFLP; act-Gal4 UAS-GFP/Cyo; tubulin-GAL80 FRT2A/TM6B)*, *hs-Flp1.22; act>FRT y+ FRT>GAL4 UAS-GFP/CyO.*

For MARCM and Flip-out clone analysis, larvae were heat shocked for 1 h at 36-42 h after egg laying (AEL), and discs were dissected and fixed at 120 h AEL. Clones were marked by the presence of GFP.

### Immunostaining and microscopy

*Drosophila* wing imaginal discs from third instar larvae were dissected in ice-cold 1× PBS (10 mM NaH_2_PO4/Na_2_HPO4, 175 mM NaCl, pH7.4) and fixed for 20 min in PBS with 4% paraformaldehyde. Fixed discs were washed three times with 0.1% Triton X-100 in PBS (PBT) and blocked in PBT containing 3% BSA for 0.5 h at room temperature. Next, discs were incubated with the primary antibodies overnight at 4°C, followed by three washes with PBT before incubating with secondary antibodies for 2 h. DAPI was added for the last 20 min. After three further washes with PBT, discs were mounted in Vectashield mounting medium (Vector Laboratories, Burlingame, CA, USA). The following primary antibodies were used: chicken anti-GFP (1:2000; ab13970, Abcam), mouse anti-Cut [1:15, 2B10, Developmental Studies Hybridoma Bank (DSHB)], mouse anti-Wingless (1:100, 4d4, DSHB), mouse anti-N^icd^ (1:100, c17.9c6, DSHB), mouse anti-N^ecd^ (1:100, c458.2h, DSHB), mouse anti-ß-galactosidase (1:1000; 23781, Promega, Madison, WI, USA) and rabbit anti-HDAC1 (1:500; J. T. Kadonaga, University of California San Diego, San Diego, CA, USA). Fluorescent secondary antibodies (Alexa Fluor 488-, 555- or 633-conjugated, anti-rabbit, anti-mouse, anti-chicken) were obtained from Molecular Probes (Waltham, MA, USA 1:500). DAPI (1 μg/ml, Sigma-Aldrich) was used to stain DNA. The images were acquired on an FV1000 confocal microscope (Olympus, Tokyo, Japan) and processed using ImageJ (https://imagej.nih.gov/ij/) and Adobe Photoshop.

### Adult wing analysis

Adult female wings were removed and mounted in 80% glycerol mounting medium. Images of wings were obtained on an Eclipse 80i microscope (Nikon, Tokyo, Japan). Wing posterior and total areas were measured with ImageJ. Statistical analysis was performed using Student's *t*-test.

## Supplementary Material

Supplementary information
