# Supplementary material for: The histone deacetylase HDAC1 positively regulates Notch signaling during Drosophila wing development
Source: Biol Open. 2018 Feb 1;7(2):bio029637. doi: 10.1242/bio.029637 (PMC5861358; doi:10.1242/bio.029637)
Supplement: Supplementary information [file biolopen-7-029637-s1.pdf]

Supplementary Figures

Figure S1 RNAi knockdown of HDAC1 reduces HDAC1 protein levels

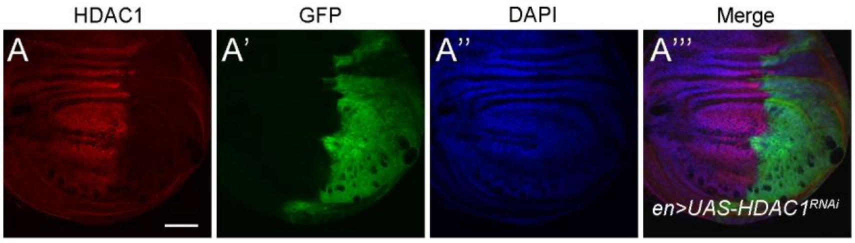

(A-A''') RNAi knockdown of *HDAC1* with *en-Gal4* reduces HDAC1 protein levels in the posterior half of the wing disc. GFP marks the expression domain of *en-Gal4*. Scale bar: 50 um.

Figure S2 RNAi knockdown of HDAC1 by various wing Gal4 drivers causes wing notching phenotype

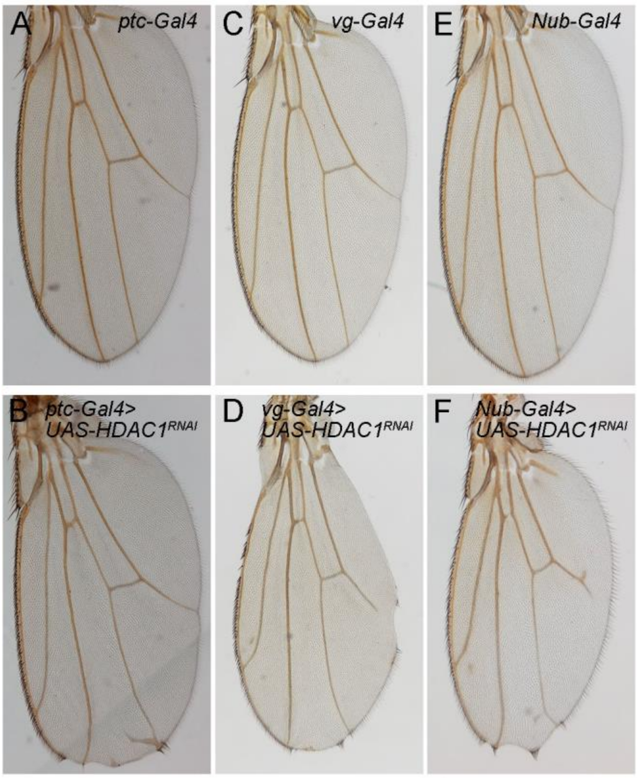

(A-C') Adult wings expressing the *UAS-HDAC1*^{RNAi} transgene under the control of *ptc-Gal4, vg-Gal4* or *nub-Gal4* display wing notches (A', B' and C'). Controls are shown in A, B and C.

Figure S3 Overexpression of HDAC1 has no obvious effects on Notch transcription and Notch target gene expression

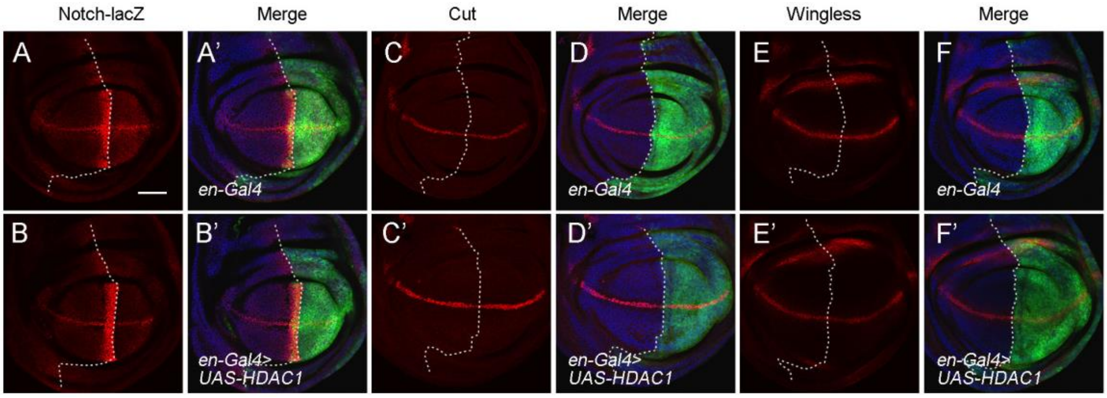

(A-B') Overexpression of *HDAC1* with *en-Gal4* does not affect *Notch-lacZ* expression in the posterior compartment of the wing disc. (C-F') Overexpression of *HDAC1* with *en-Gal4* does not affect Cut (C-D') and Wingless (E-F') protein levels in the posterior half of the wing disc. GFP marks the expression domain of *en-Gal4*. Scale bars: 50 um.
